# GABARAP suppresses EMT and breast cancer progression via the AKT/mTOR signaling pathway

**DOI:** 10.18632/aging.202510

**Published:** 2021-02-11

**Authors:** Ying Liu, Dandan Wang, Mengxia Lei, Jiayi Gao, Yuqing Cui, Xiaoying Jin, Qiujie Yu, Ying Jiang, Yan Guo, Yali Liu, Li Cai, Xuesong Chen

**Affiliations:** 1 The Fourth Department of Medical Oncology, Harbin Medical University Cancer Hospital, Harbin 150040, China; 2 Radiology Department of Medical Oncology, Harbin Medical University Cancer Hospital, Harbin 150040, China; 3 Department of Biochemistry and Molecular Biology, Mudanjiang Medical University, Mudanjiang 157000, China

**Keywords:** GABARAP, breast cancer, EMT, prognosis, AKT/mTOR pathway

## Abstract

Few studies have focused on γ-aminobutyric acid type A (GABA_A_) receptor-associated protein (GABARAP) in tumor progression. We investigated the expression and importance of GABARAP in breast cancer. We analyzed the expression of GABARAP and its relationship with clinicopathological features and prognosis (TCGA). To explain the role and potential mechanism of GABARAP in regulating tumor development, we performed acquisition and loss of function experiments using cell lines and models of mouse xenotransplantation. We found that GABARAP inhibited proliferation, migration and invasion *in vitro* and *in vivo*. Notably, low levels of GABARAP induced the epithelial-mesenchymal transition (EMT). Low levels of GABARAP increased p-AKT and p-mTOR levels, and a specific AKT pathway inhibitor reversed the downregulation of GABARAP-induced tumor progression. GABARAP negatively correlated with advanced clinicopathological features in clinical specimens, such as tumor size and TNM stage. Notably, patients with low GABARAP levels had a poor prognosis. Immunohistochemistry (IHC) revealed that GABARAP expression negatively correlated with matrix metalloproteinase (MMP) 2 and MMP14. Conclusively, these data indicate that GABARAP suppresses the malignant behaviors of breast cancer likely via the AKT/mTOR pathway. The targeting of GABARAP may improve the certainty of diagnosis and treatment strategies for breast cancer.

## INTRODUCTION

Breast cancer is the most common cancer in women worldwide, and it ranks second among deaths from cancer [1]. The number of new breast cancer cases has continually risen in recent years in China, and the age of onset tends to be younger [2]. A dismal prognosis is expected in patients with aggressive tumors that are refractory to various treatments, and a large number of patients die from complications associated with metastatic diseases [3, 4]. Metastasis and chemoresistance lead to treatment failure, and the mechanisms responsible are poorly understood. Therefore, the molecular mechanisms underlying breast cancer progression must be further characterized, and novel mechanism-based therapeutic strategies must be developed to block this process [5, 6]. The present study assessed the possible value of γ-aminobutyric acid type A (GABA_A_) receptor-associated protein (GABARAP) as a therapeutic target in the metastasis of breast cancer.

GABARAP is a 14-kDa cytoplasmic protein located at 17p13.1 that plays a vital role in regulating GABA_A_ receptor activity and intracellular trafficking [7, 8]. GABARAP binds to intracellular proteins that are generally related with vesicle transport, autophagy and apoptosis, including the cytoskeleton, tubulin, puerarin, tretin heavy chain, phospholipase C-related, but catalytically inactive protein (PRIP), namely p130/phospholipase C-related inactive protein, transferrin receptor, Unc-51-like kinase, RAS-associated protein 24, and angiotensin II type 1 (AT1) receptor protein [9–14]. Although recent reports showed that GABARAP had a profound impact on the regulation of inflammatory progression [15] and angiogenic activity [16], its expression and mechanism in breast cancer are not clear, which prompted investigation of the significant role of GABARAP in the occurrence and growth of breast cancer.

The epithelial-mesenchymal transition (EMT) is a pivotal and complex mechanism for the invasiveness of various epithelial tumors [17, 18], and it primarily involves multiple signals from the tumor microenvironment, tumor cells and the interaction between these two components [19–21]. The PI3K/AKT signaling pathway profoundly impacts cell proliferation, adhesion, migration, invasion, metabolism and survival. AKT also plays a vital role in the development of tumors and may be target for the treatment of the cancer because it is over active in greater than 60% the above-mentioned cancers [22–24]. We used a comprehensive research method based on clinical breast cancer specimens, cell and animal models to examine the expression and biological function of GABARAP. We also elucidated the mechanism of GABARAP in the inhibition of EMT in breast cancer cells, which is partially dependent on the AKT/mTOR signaling pathway.

## RESULTS

### GABARAP is downregulated in primary breast cancer specimens and breast cancer cell lines

**Figure 1:**
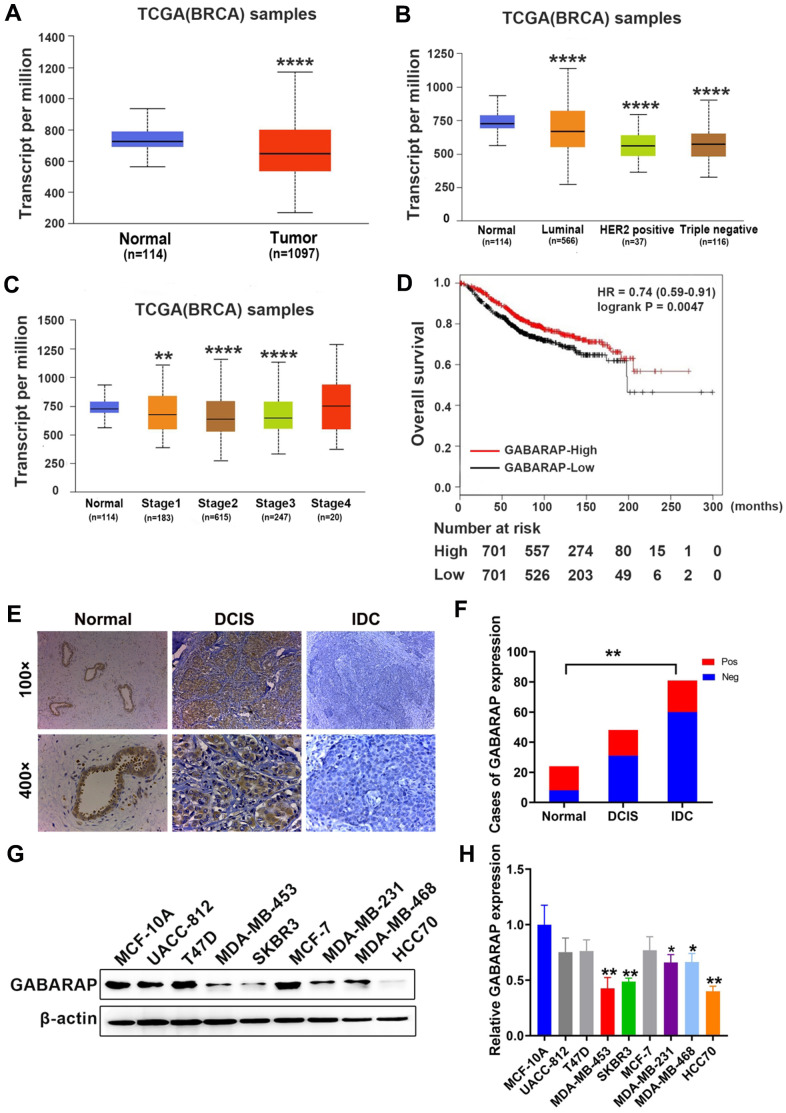
**GABARAP is downregulated in breast cancer specimens and cell lines.** (**A**) Expression profile of GABARAP in primary breast cancer tissues (n = 1097) and normal breast tissues (n = 114) (TCGA). (**B**) Expression of GABARAP in BRCA based on breast cancer subclasses (TCGA). (**C**) Expression of GABARAP in BRCA based on individual cancer stages (TCGA). (**D**) Overall survival of breast cancer patients according to GABARAP expression (TCGA). (**E**) Representative images of GABARAP immunohistochemical staining. (magnification, 100× and 400×). (**F**) Statistical significance of ‘positive’ or ‘negative’ GABARAP staining in 87 cases of IDC, 48 cases of DCIS and 24 cases of normal breast tissue. (**G**) Western blotting analysis of GABARAP expression in 8 human breast cancer cell lines and non-transformed MCF-10A cells. (**H**) Statistical significance of GABARAP expression in 8 human breast cancer cell lines and non-transformed MCF-10A cells. (*P < 0.05; **P < 0.01; ***P < 0.001; ****P < 0.0001. TCGA = The Cancer Genome Atlas).

The analyses of GABARAP mRNA expression in the breast cancer RNA sequence data in TCGA database strongly support the study of its potential role in breast cancer. According to the results, GABARAP mRNA expression in breast cancer tissues was much lower than normal tissue (*P* < 0.0001; Figure 1A), and its expression in breast cancer subtypes was also lower (*P* < 0.0001; Figure 1B). We also found that GABARAP mRNA negatively correlated with the clinical phase of breast cancer (*P* < 0.01; Figure 1C). Survival analyses revealed that breast cancer patients with low expression of GABARAP had a shorter survival time (*P* = 0.0047; Figure 1D). IHC validated the results of the TCGA data analysis of 87 IDC samples, 48 DCIS samples and 24 non-cancer tissue samples (Figure 1E). Positive staining (brown) was detected in most non-cancerous tissues (16 / 24) and some DCIS tissues (17 / 48), but the number of positively stained cells in IDC tissues was relatively small (27 / 87) (*P* < 0.01; Figure 1F, Supplementary Table 1). These results indicate that the low expression of GABARAP may be related to the occurrence and development of breast cancer.

To examine whether GABARAP was also decreased in the cultured breast cancer cell lines, we performed Western blot analysis of eight breast cancer cell lines and non-transformed MCF-10A cells. As shown in Figure 1G and 1H, GABARAP expression levels were high in the non-transformed MCF-10A cells, weak in the T47D, UACC-812, and MCF-7 cells and low in the MDA-MB-453, SKBR3, HCC70, MDA-MB-231 and MDA-MB-468 cells (*P*<0.05). These results indicate that GABARAP is downregulated in breast cancer cells.

### Low GABARAP levels enhance the malignant behavior of breast cancer cells

**Figure 2:**
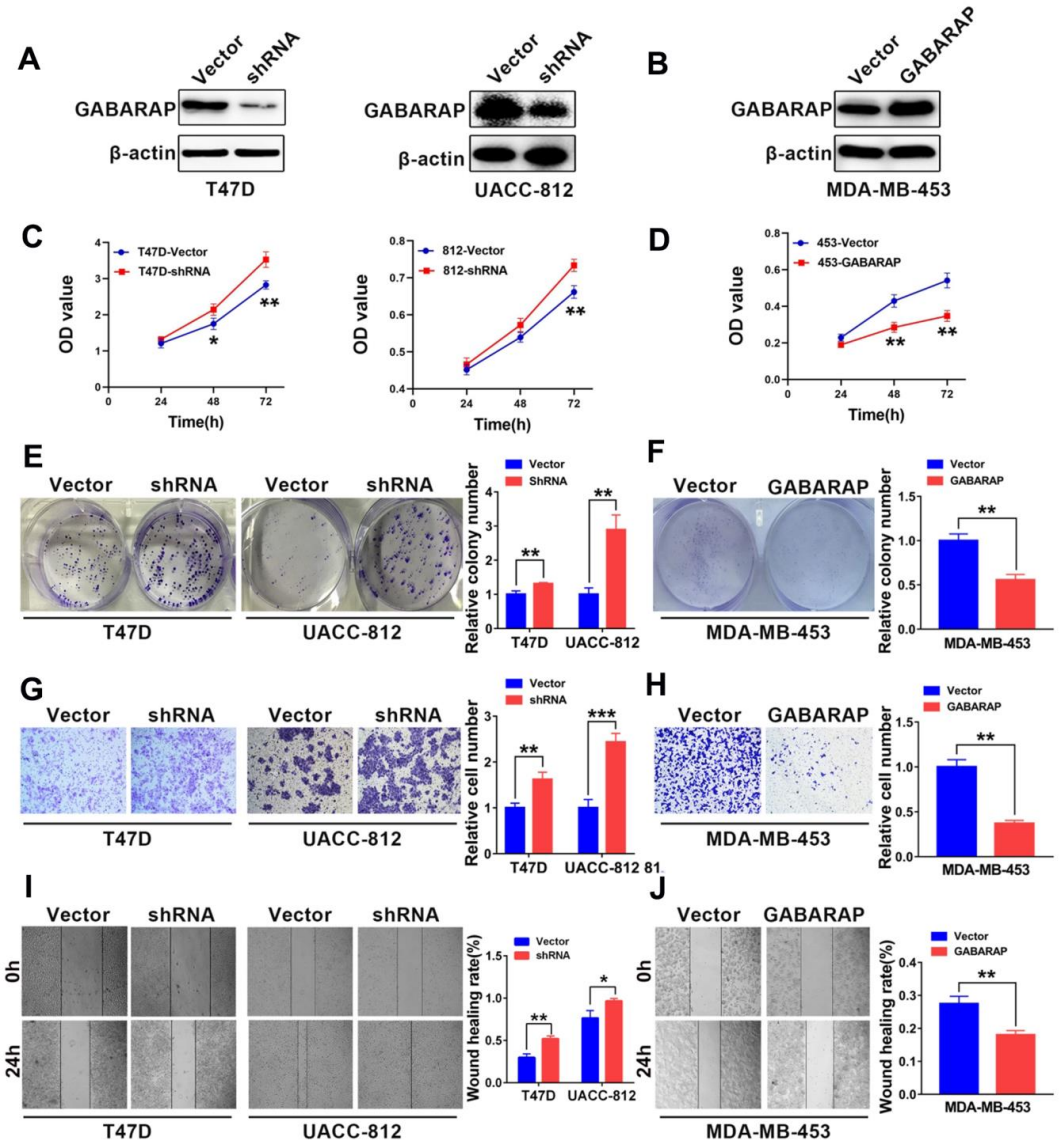
**GABARAP suppresses the malignant behavior of breast cancer cells.** (**A**) Knockdown of GABARAP in T47D and UACC-812 cells; GABARAP expression was determined using Western blot. (**B**) Overexpression of GABARAP in MDA-MB-453 cells; GABARAP expression was determined using Western blot. (**C**) Cell proliferation in T47D-vector, T47D-shRNA, UACC-812-vector and UACC-812-shRNA cells was detected using CCK-8 assays. (**D**) Cell proliferation in MDA-MB-453-vector and MDA-MB-453-GABARAP cells was detected using CCK-8 assays. (**E**) Colony-forming efficiency was determined in T47D-vector, T47D-shRNA, UACC-812-vector and UACC-812-shRNA cells. (**F**) Colony-forming efficiency was determined in MDA-MB-453-vector and MDA-MB-453-GABARAP cells. (**G**) Invasive abilities of T47D-vector, T47D-shRNA, UACC-812-vector and UACC-812-shRNA cells were measured using Matrigel invasion assays. (**H**) Invasive abilities of MDA-MB-453-vector and MDA-MB-453-GABARAP cells were measured using Matrigel invasion assays. (**I**) Migration abilities of T47D-vector, T47D-shRNA, UACC-812-vector and UACC-812-shRNA cells were assessed using wound-healing migration assays. (**J**) Migration abilities of MDA-MB-453-vector and MDA-MB-453-GABARAP cells were assessed using wound-healing migration assays. Experiments were performed at least three times. The data are expressed as the means ± SEM. *P* values were calculated using Student’s t-test. (*P < 0.05; **P < 0.01; ***P < 0.001).

We used Western blotting analysis to compare the expression levels of GABARAP in the breast cancer cell lines and non-transformed MCF-10A cells. As shown in Figure 1G, the T47D and UACC-812 cells were used as GABARAP “loss-of-function” models, and the MDA-MB-453 cells served as a GABARAP “gain-of-function” model. GABARAP-related protein expression levels in these target cells were detected using Western blot. As shown in Figure 2A, the T47D and UACC-812 cells exhibited significant knockdown of GABARAP compared to the control cells, and the MDA-MB-453 cells exhibited an upregulation of GABARAP (Figure 2B).

To examine whether GABARAP influenced the proliferation of breast cancer cell lines, we performed CCK8 assays to measure cell viability. GABARAP knockdown severely increased cell growth in the T47D and UACC-812 cells compared to the respective control cells (Figure 2C), and the overexpression of GABARAP attenuated cell growth in the MDA-MB-453 cells (Figure 2D). We comparatively observed similar patterns in the efficiency of the colony formation of GABARAP-shRNA-transfected T47D and UACC-812 cells (Figure 2E) and GABARAP-overexpressing MDA-MB-453 cells (Figure 2F), which suggests that GABARAP negatively regulates cell proliferation *in vitro*.

We investigated the possible role of GABARAP in cell migration and invasion using the wound healing and Transwell assays, respectively. Cell proliferation may affect the results of the Transwell assays. We used the respective cell proliferation rates to normalize the number of invaded cells and evaluated the invasive ability of breast cancer cells. As shown in Figure 2G–2J, migration and invasion were significantly enhanced in T47D and UACC-812 cells with knockdown of GABARAP. However, the migration and invasion of cells were markedly inhibited in MDA-MB-453 cells with GABARAP overexpression compared to MDA-MB-453 cells transfected with vector controls. These results show that GABARAP inhibits cell migration and invasion *in vitro*.

### GABARAP may regulate breast cancer progression via the EMT

**Figure 3:**
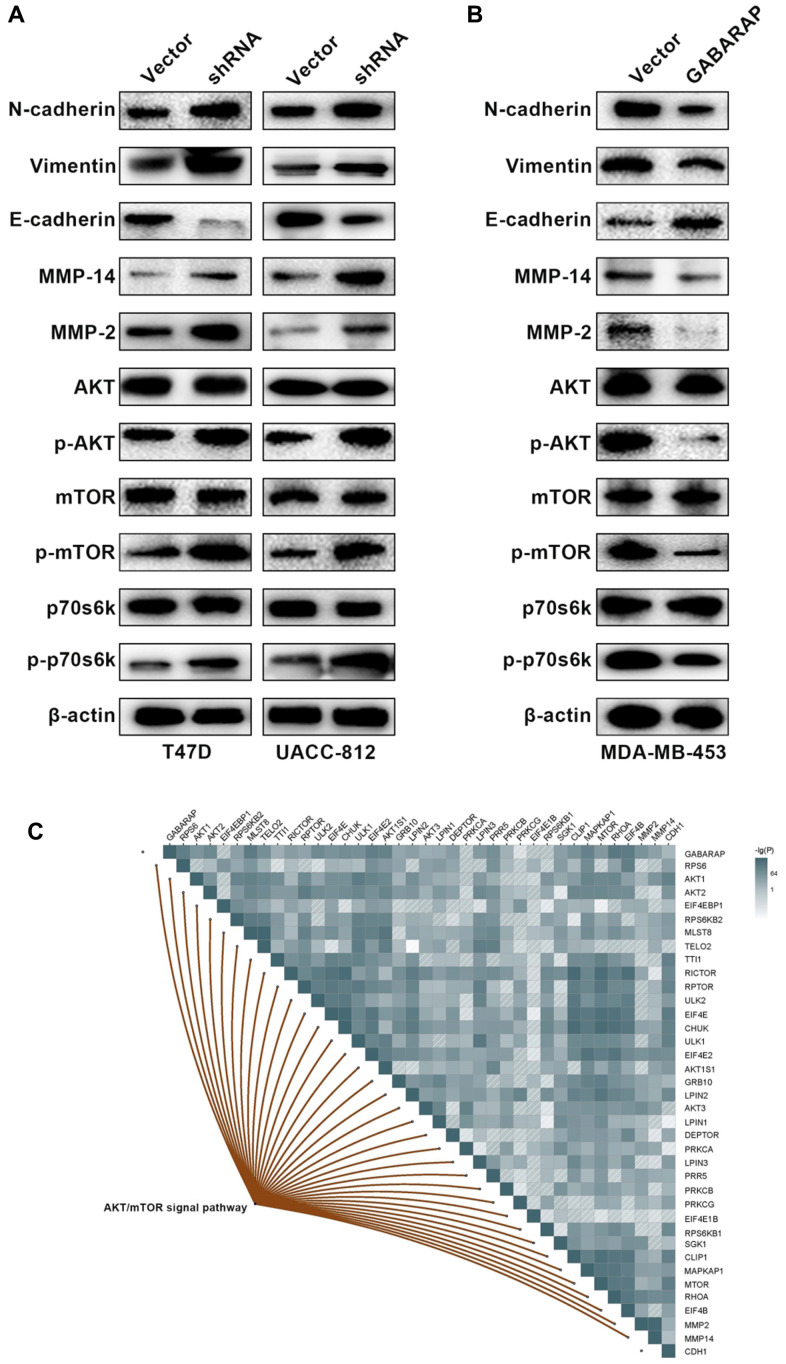
**Low GABARAP level promotes cellular EMT via AKT/mTOR signaling in breast cancer.** (**A**) Western blot analyses were used to detect the expression levels of E-cadherin, N-cadherin, vimentin, MMP2, MMP14, p-AKT, AKT, p-mTOR, mTOR, p-p70s6k and p70s6k in T47D-vector, T47D-shRNA, UACC-812-vector and UACC-812-shRNA cells. Cells were lysed using RIPA lysis buffer containing protease inhibitors and a phosphorylase inhibitor cocktail to obtain protein. β-actin was used as an internal control. (**B**) Western blot analyses were used to detect the expression levels of E-cadherin, N-cadherin, vimentin, MMP2, MMP14, p-AKT, AKT, p-mTOR, mTOR, p-p70s6k and p70s6k in MDA-MB-453-vector and MDA-MB-453-GABARAP cells. Cells were lysed using RIPA lysis buffer containing protease inhibitors and a phosphorylase inhibitor cocktail to obtain protein. β-actin was used as an internal control. (**C**) Pearson correlation was calculated among genes related to GABARAP, CDH1, MMP2, MMP14 and the AKT/ mTOR signaling pathway in breast cancer patients clinical cohort (TCGA).

The EMT plays a pivotal role in tumor metastasis, and we hypothesized that GABARAP affected the EMT process and inhibited breast cancer progression. When EMT occurs, the expression of various marker proteins changes, and detecting the expression of EMT markers is the main method to verify the occurrence of EMT in tissue cells. Therefore, Western blot was used to detect the expression changes in E-cadherin, N-cadherin, vimentin, MMP2 and MMP14 in each cell model group. Western blots demonstrated that the downregulation of GABARAP was associated with high levels of vimentin, N-cadherin, MMP2 and MMP14 and low levels of E-cadherin (Figure 3A). Conversely, GABARAP upregulation inhibited vimentin, N-cadherin, MMP2 and MMP14 expression and upregulated E-cadherin expression (Figure 3B). These results indicate that GABARAP inhibits the EMT and suppresses breast cancer progression.

### Low levels of GABARAP induces the EMT by activating the AKT/mTOR pathway

**Figure 4:**
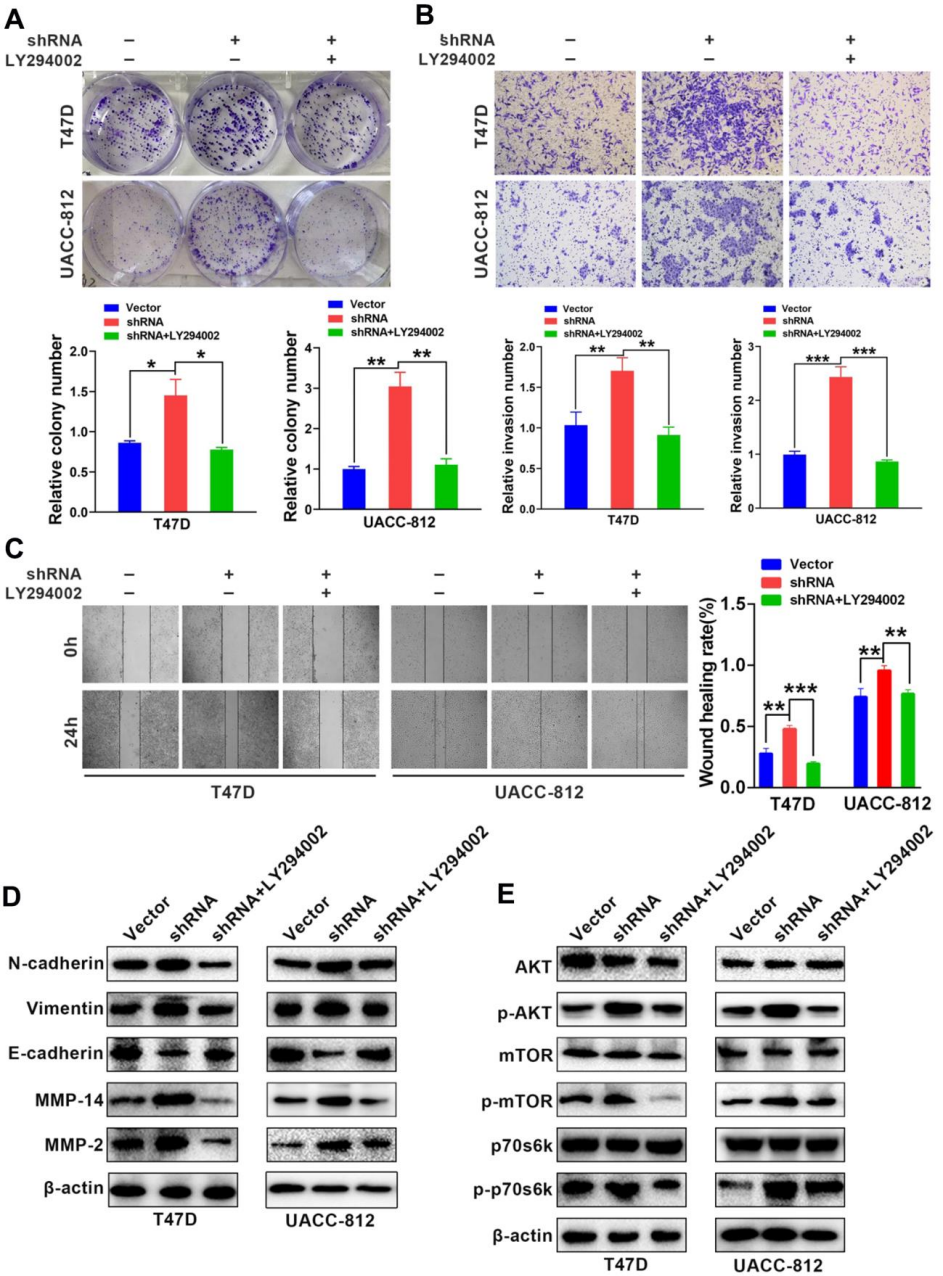
**LY-294002 (AKT pathway inhibitor) reverses GABARAP-inhibited proliferation, invasion, migration and EMT.** (**A**) Colony-forming efficiency was assessed in T47D-Vector, T47D-shRNA, T47D-shRNA cells incubated with LY-294002, UACC-812-vector, UACC-812-shRNA, and UACC-812-shRNA cells incubated with LY-294002. *P* values were calculated using Student’s t-test. (**B**) Invasion assays were performed in the indicated cells. *P* values were calculated using Student’s t-test. (**C**) Migration assays were performed in the indicated cells. *P* values were calculated using Student’s t-test. (**D**) Western blot analyses were used to detect the expression levels of E-cadherin, N-cadherin, vimentin, MMP2 and MMP14 in the indicated cells. β-actin was used as an internal control. (**E**) Western blot analyses were used to detect the expression levels of p-AKT, AKT, p-mTOR, mTOR, p-p70s6k and p70s6k in the indicated cells. Cells were lysed using RIPA lysis buffer containing protease inhibitors and a phosphorylase inhibitor cocktail to obtain protein. β-actin was used as an internal control. Experiments were performed at least three times. The data are expressed as the mean ± SEM. *P* values were calculated using Student’s t-test. (*P < 0.05; **P < 0.01; ***P < 0.001; ****P < 0.0001).

The AKT/mTOR, NF-κB and ERK/MAPK signaling pathways are the main regulatory pathways of tumor EMT [25–27]. GABARAP regulates a number of classic signaling pathways, including the AKT/mTOR pathway [28]. Therefore, we examined whether GABARAP inhibited the EMT of breast cancer via regulation of the AKT/mTOR pathway. We calculated and analyzed the correlation between GABARAP and the AKT/mTOR pathway-related genes using bioinformatics software and concluded that GABARAP significantly correlated with the AKT/mTOR pathway (Figure 3C). Western blotting confirmed that the downregulation of GABARAP in T47D and UACC-812 cells increased the levels of p-AKT, p-mTOR and p-p70s6k (Figure 3A), but it did not significantly change the expression of p-ERK, p-MEK, p-IKK-β or p-IκBα (Supplementary Figure 1A). The overexpression of GABARAP suppressed the phosphorylation of AKT, mTOR and p70s6k (Figure 3B) but showed no effect on p-ERK, p-MEK, p-IKK-β or p-IκBα levels (Supplementary Figure 1B). These results demonstrated that GABARAP levels were negatively related to the activation of the AKT/mTOR pathways. We used pathway inhibitors and found that GABARAP-mediated inhibition of invasion, migration, and the EMT was reversed by the AKT pathway inhibitor LY-294002 (50 μM, 24 h; Figure 4A–4E). These data demonstrate that the downregulation of GABARAP activated the AKT/mTOR pathway to promote EMT in breast cancer cells.

### GABARAP suppresses breast cancer progression *in vivo*

**Figure 5:**
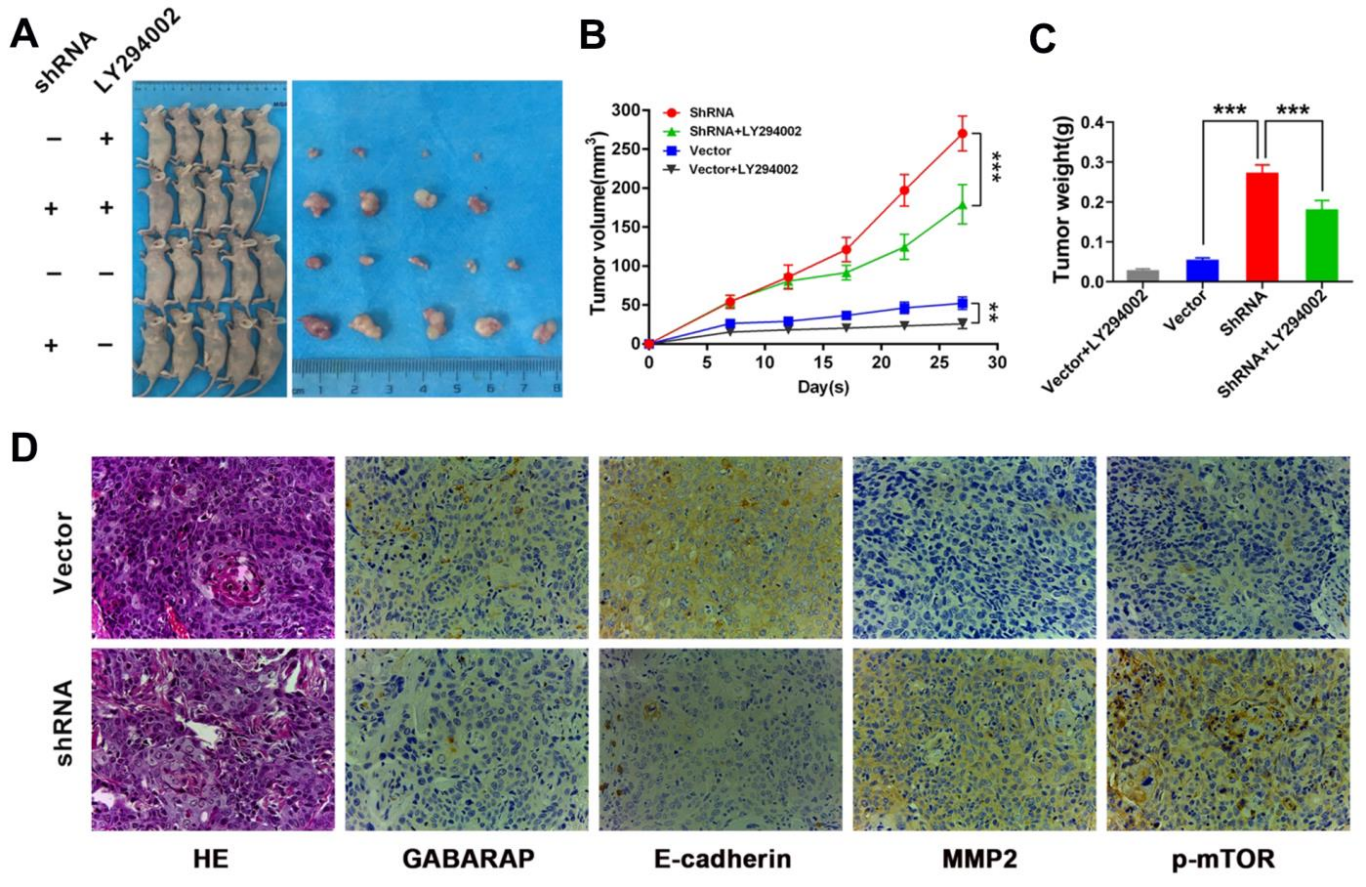
**GABARAP suppresses breast cancer progression *in vivo*.** (**A**) A total of 5×10^6^ GABARAP-knockdown or control cells were injected subcutaneously into the left side of each of nude mouse. Vector group, mice inoculated with control UACC-812 cells; shRNA group, mice inoculated with GABARAP silenced UACC-812 cells; vector control+LY294002 group, mice inoculated with control UACC-812 cells and treated with LY294002; shRNA+LY294002 group, mice inoculated with GABARAP silenced UACC-812 cells and treated with LY294002. Representative images of nude mice and tumors at day 28 after inoculation of UACC-812 cells with or without shRNA-mediated silencing of GABARAP. (**B**) Tumor growth curves in 4 groups of nude mice. The data are presented as the means ± SDs. *P* values were calculated using Student’s t-test. (**C**) The tumor weights were measured. The data were statistically analyzed using Student’s t-test, and the mean ± SEM is shown. (**D**) Immunostaining of proteins in tumors from the vector control group and GABARAP-shRNA group. First column, H&E staining; second column, immunostaining for GABARAP; third column, immunostaining for E-cadherin; fourth column, immunostaining for MMP2; and fifth column, immunostaining for p-mTOR. Magnification, 400×. (*P < 0.05; **P < 0.01; ***P < 0.001).

To evaluate the role GABARAP in the progress of breast cancer *in vivo*, we constructed a xenograft tumor model in nude mice using UACC-812 cell lines steadily transfected with vector control or GABARAP-shRNA. The animals were treated with LY294002 (75 mg/kg) or sterile water. Mice were assigned at random to the following experimental groups: vector control group, GABARAP-shRNA group, vector control + LY294002 group and GABARAP-shRNA + LY294002 group (n=5 per subgroup). (Figure 5A). The silencing of GABARAP significantly increased the tumor volumes and weights compared to control. However, LY-294002 reversed GABARAP-mediated inhibition of tumor formation (Figure 5B, 5C). Tumors with GABARAP knockdown had a higher metastasis capacity, as evidenced by the increased MMP2 and p-mTOR staining and reduced E-cadherin staining (Figure 5D). These results indicated that the knockdown of GABARAP suppressed breast cancer progression *in vivo*.

### Clinical significance of GABARAP in patients with breast cancer

Pathology results from the Harbin Medical University Cancer Center (HMUCC) were used to investigate the correlation between GABARAP level and the clinicopathological characteristics of 87 IDC patients. As shown in Supplementary Table 2, statistical analyses of IHC results showed that low levels of GABARAP were associated with advanced pT grade, axillary lymph node metastasis, advanced pTNM stage, histological grade and ER status (P values of 0.025, 0.023, 0.001, 0.019 and 0.039, respectively). However, whether it was associated with age, HER-2 status, Ki-67 level or p53 status was not clear. The final results indicated that GABARAP was related to the clinicopathological characteristics of malignant tumors. Therefore, we speculate that low GABARAP expression will affect the proliferation of tumor cells and plays a profound role in the occurrence and growth of breast cancer.

### GABARAP expression correlates with MMP2 and MMP14 expression in human breast cancer specimens

**Figure 6:**
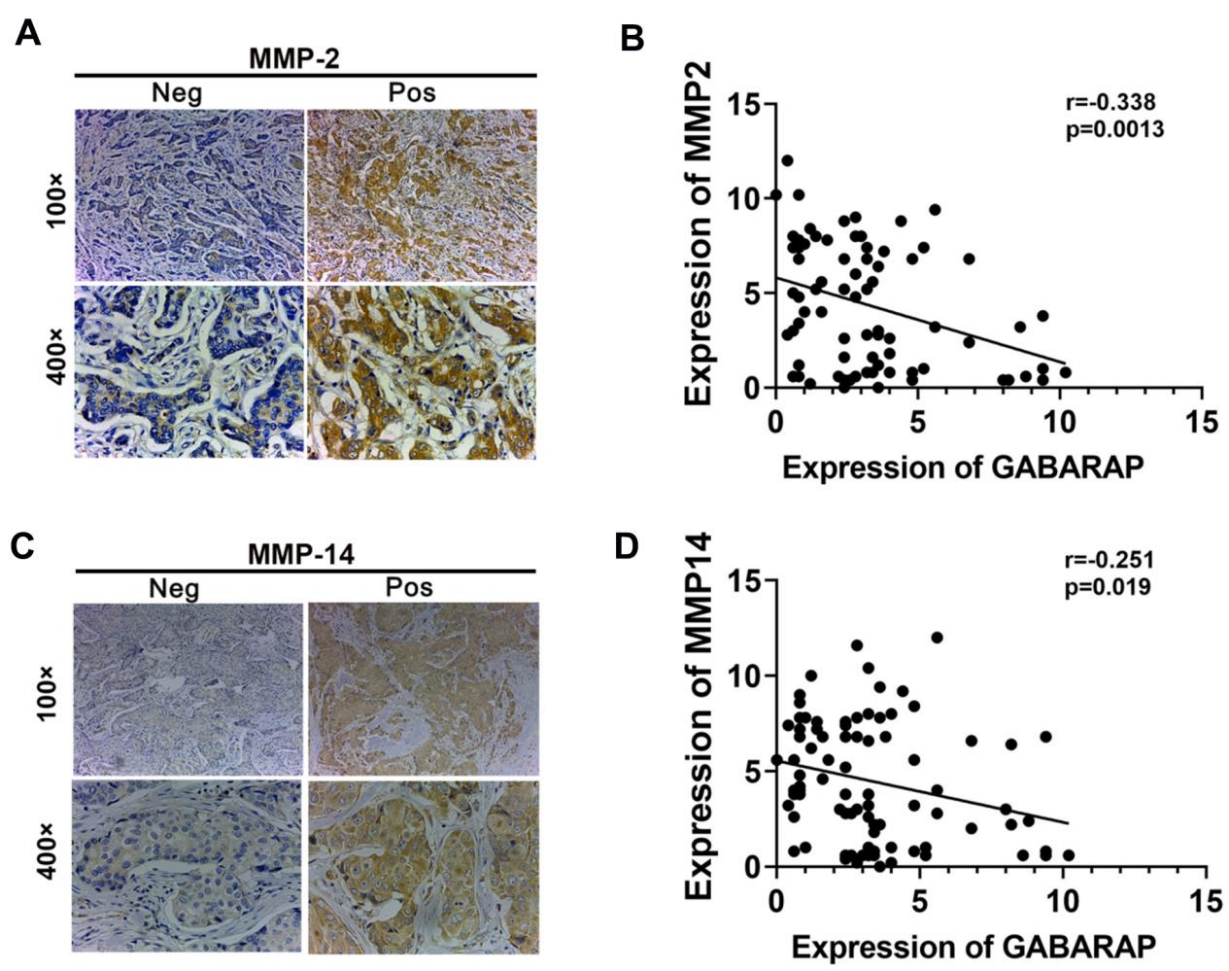
**Correlation between MMPs and GABARAP expression in breast cancer tissues from patients.** (**A**) Representative immunostaining profiles of MMP2 in GABARAP low expression and GABARAP high expression breast cancer tissues. Magnification, 100× and 400×. (**B**) Correlation analysis of the expression of MMP2 and GABARAP using the Pearson correlation coefficient. GABARAP negatively correlated with MMP2 at the protein level. (**C**) Representative immunostaining profiles of MMP14 in GABARAP low expression and GABARAP high expression breast cancer tissues. Magnification, 100× and 400×. (**D**) Correlation analysis of the expression of MMP14 and GABARAP using the Pearson correlation coefficient. GABARAP negatively correlated with MMP14 at the protein level.

To obtain a deeper understanding of the relationship between GABARAP and metastasis in human breast cancer, IHC staining of GABARAP, MMP2 and MMP14 was performed in 87 IDC specimens (Figure 6A–6C). Consistent with the observations of the tumor cell lines and xenograft models, the distribution and intensity of GABARAP negatively correlated with MMP2 (*P*=0.0013; Figure 6B) and MMP14 (*P*=0.019; Figure 6D) in breast cancer tissue specimens. This finding clearly indicated that low GABARAP expression was related with elevated metastasis in breast cancer patients.

## DISCUSSION

Metastasis is the main cause of treatment failure in breast cancer patients [29, 30], and the EMT is an important mechanism of tumor metastasis. Few reports focused on GABARAP and EMT-related tumor metastasis. Our team demonstrated, for the first time, that GABARAP inhibited EMT-related breast cancer tumor progression in a cell model, an animal model and human breast cancer tissue samples, and the mechanism may involve direct regulation of the AKT/mTOR pathway.

To investigate the expression and role of GABARAP in breast cancer, we first analyzed GABARAP mRNA expression using RNA-seq data from the TCGA breast cancer cohort. Compared to normal tissue, the expression of GABARAP was extremely downregulated. Compared to normal tissues, the expression of GABARAP in any subtype of breast cancer was lower, and gradually decreased with increasing clinical stage from stage I to III. Survival analysis revealed that the survival time of breast cancer patients with low expression of GABARAP was shorter. These results suggest that GABARAP is related to the occurrence and growth of breast cancer. The expression of GABARAP in normal breast tissue, intraductal carcinoma and invasive ductal carcinoma was detected using IHC. The results demonstrated that the expression of GABARAP gradually declined with the increase of tumor malignancy. This conclusion is consistent with publicly available data, which suggest that GABARAP may be used as a prognostic predictor of this type of cancer.

Our study performed an experimental validation at the cellular level on two GABARAP-high cell lines (UACC-812 and T47D) and one GABARAP-low breast cancer cell line (MDA-MB-453). The different cell lines, UACC-812 (ER-, PR-, HER2+), T47D (ER+, PR+/-, HER2-) and MDA-MB-453 (ER-, PR-, HER2+), represented different subtypes of breast cancer, and these subtypes have different biological, clinical and molecular characteristics. Therefore, we selected these three cell lines and performed functional experiments on different breast cancer subtypes. The results showed that the downregulation of GABARAP promoted the proliferation, invasion and metastasis of the different subtypes, and the overexpression of GABARAP inhibited the proliferation and metastasis of breast cancer cells.

EMT is one of the main mechanisms leading to tumor invasion and metastasis [31, 32]. Therefore, we examined whether GABARAP inhibited tumor cell migration and invasion via regulation of the EMT in breast cancer cells. Western blotting was used to detect the expression of E-cadherin, N-cadherin, vimentin, MMP2 and MMP14 in different functional models of breast cancer subtypes to verify the occurrence of EMT. The results demonstrated that the overexpression of GABARAP suppressed the expression of stromal markers, such as vimentin, N-cadherin, MMP2 and MMP14, and promoted the expression of epithelial markers, such as E-cadherin. The expression of vimentin, N-cadherin, MMP2 and MMP14 was increased in the T47D-shRNA and UACC-812-shRNA groups, and the expression of E-cadherin was inhibited. These results demonstrated that GABARAP inhibited the proliferation and metastasis of breast cancer cells via the regulation of EMT. Previous studies also reported the role of GABARAP in tumor autophagy, drug resistance and apoptosis [33, 34]. Our study first revealed the role of GABARAP in the invasion and metastasis of breast cancer. As a negative regulator, GABARAP inhibits the activities of breast cancer via regulation of the EMT, which establishes the value and significance of GABARAP as an underlying target for therapies of the multi-level progression of the specific cancer.

Multiple signaling pathways, including the mTOR, PI3K/AKT and NF-κB pathways, are involved in GABARAP-regulated autophagy, inflammation and angiogenic activity [8–13]. Wu et al. reported that GABARAP promoted bone marrow mesenchymal stem cell-mediated osteoarthritis cartilage regeneration via inhibition of the PI3K/AKT/mTOR signaling pathway [28]. The downregulation of GABARAP in T47D and UACC-812 cells in our study increased the levels of p-AKT, p-mTOR and p-p70s6k. An inhibitor of the AKT/mTOR pathway, LY-294002, reversed the proliferation and invasion of breast cancer induced by GABARAP knockdown. These results demonstrated that GABARAP inhibited the invasion and metastasis of breast cancer by regulating the EMT via downregulation of the AKT/mTOR pathway.

We constructed an orthotopic tumor model in nude mice *in vivo*, and the experimental results showed that GABARAP inhibited the growth of tumors *in vivo*, which is consistent with the relevant results. The IHC-related outcome showed that the expression of E-cadherin was greatly decreased, and MMP2 and p-mTOR expression was increased by the knockdown of GABARAP. The results indicated that GABARAP also inhibited the EMT of breast cancer cells via the AKT/mTOR pathway *in vivo*. This conclusion confirmed the *in vitro* experimental findings. The expression patterns observed *in vivo* also confirmed that GABARAP did not have a one-to-one regulatory relationship with the AKT target. Therefore, other cell factors are involved in the regulation of the AKT/mTOR pathway, and GABARAP may also regulate other pathways in addition to the AKT/mTOR pathway. For example, Wei et al. reported that TRIM44 also activated the AKT/mTOR signaling pathway to induce melanoma progression and stabilize TLR4 [35]. García et al. showed that GABARAP regulated Rho signaling to suppresses skin tumor formation and invasion [10]. Therefore, we conclude that low levels of GABARAP lead to partial activation of the AKT/mTOR signaling pathway.

In human breast cancer tissue samples, we first analyzed the relationship between GABARAP and the clinicopathology-related features of breast cancer. The results showed that GABARAP was related to malignant clinicopathological characteristics, which is consistent with publicly available data. The expression of MMP2 and MMP14 in human breast cancer samples was detected using IHC, and the results showed that GABARAP negatively correlated with MMP2 and MMP14, which indicates that GABARAP also inhibits invasion and metastasis in human breast cancer samples.

Recent studies reported that GABARAP plays a vital role in the level of autophagy. For example, Sasai M et al. demonstrated that GABARAP was an autophagy protein and played an essential role in interferon-inducible GTPase-mediated host defense [36]. Previous papers also described the function of autophagy in the regulation of the EMT. For example, Gugnoni M et al. previously demonstrated that CDH6 interacted with GABARAP to promote the EMT and thyroid tumor metastasis by restraining autophagy [37]. Akalay I et al. indicated that the EMT and autophagy induction in breast carcinoma promoted the escape from T-cell-mediated lysis [38]. However, whether GABARAP restrained the EMT and whether breast cancer development was involved in the autophagy process were not determined and will be a focus of our future research.

In conclusion, we demonstrated that GABARAP suppressed the proliferation and invasion of breast cancer cells via regulation of the EMT *in vitro* and *in vivo*, and the mechanism may be related to regulation of the AKT/mTOR pathway. Evaluation of breast cancer patients and clinical data indicated that GABARAP was associated with the clinicopathology-related characteristics of malignancy and negatively correlated with the expression of MMP2 and MMP14. Therefore, our results show that GABARAP represents a considerable target for breast cancer treatment and a new prognostic indicator.

## MATERIALS AND METHODS

### Tissue specimens and patients

The Ethics Committee of Harbin Medical University approved the study. Eighty-seven IDC (invasive ductal carcinoma) cases, 48 DCIS (ductal carcinoma *in situ*) cases and 24 cases of normal tissue were selected. The IDC patients were female and were hospitalized in the Affiliated Cancer Hospital of Harbin Medical University between March 2010 and November 2010. The patients were followed up until March 2015. Overall, the mean follow-up time was 58.9 months (16.8–63.3 months). Formalin was used to fix the paraffin-embedded tissues of the selected cases, and the complete clinical records were obtained. None of the patients received chemotherapy or radiotherapy before surgery.

### Cell culture

Except the human breast cancer cell line MDA-MB-231, MCF7, MDA-MB-468, T47D, UACC-812, MDA-MB-453, SKBR-3, and HCC70 cells, and the non-transformed breast cell line MCF-10A were secured from the Cancer Research Institute of Heilongjiang Province. MCF7, T47D, UACC-812 and HCC70 cells were cultured in DMEM (Gibco, Carlsbad, CA, USA), and MDA-MB-453 and SKBR-3 cells were cultured in RPMI 1640 (Gibco). All of media for the cancer cell lines were supplemented with 10% fetal bovine serum (FBS) and 1% penicillin-streptomycin from (Gibco, NY, USA). MCF-10A cells were cultured in DMEM-F12 (Gibco, Carlsbad, CA, USA) medium supplemented with 0.5 μg/ml hydrocortisone, 10 μg/ml insulin, 20 ng/ml hEGF and 10% FBS. All cells were placed in a humidified incubator at 37° C with 5% CO2. MDA-MB-231 and MDA-MB-468 cells were cultured in RPMI 1640 (Gibco, Carlsbad, CA, USA) supplemented with 10% FBS and 1% penicillin-streptomycin at 37° C without CO2.

### Cell transfection

UACC-812 and T47D cells were infected with lentiviruses expressing specific shRNA to knock down GABARAP (GABARAP-shRNA). Human GABARAP-targeted RNAi (RNAi: GCCUACAG UGACGAAAGUGTT) sequences were obtained from GeneChem Co. Ltd. (Shanghai, China). Mixed versions of these sequences (NC: GGCUCUAGAAAAGCCUAUGCdTdT) were used as a control. For overexpression of GABARAP in MDA-MB-453 cells, the fragment containing the GABARAP coding sequence was subcloned into the pcDNA3.1 vector between the EcoRI and HindIII sites. The GABARAP sequence, was synthesized by Shanghai GeneChem Co. Ltd. according to NCBI Reference Sequence (NM_007278.2). Briefly, these cells were infected by lentivirus, and the steady cell lines were formed. After 24 hours, the cells were transferred into the media containing 4 μg/ml puromycin for 3 days.

### Cell viability assay

The proliferation of T47D, UACC-812 and MDA-MB-453 cells was assessed using a CCK (Cell Counting Kit)-8 (Shanghai Beyotime Institute of Biotechnology) according to the manufacturer’s instructions. Depending on cell type, 1000–3000 cells per well were cultured in 96-well plates, which was fortified with CCK-8 reagent after 24 h, 48 h and 72 h, with 10 μl per well. After incubation at 37° C for 2 h, the optical density (OD) absorbance at 450 nm was measured. Each analysis was based on three parallel experiments, and there were five repeating wells for each condition.

### Cell invasion assays

We inoculated cell (5 × 10^4^ - 1× 10^5^) suspensions (200 μl of serum-free medium) into 8-mm Pore Transwell Inserts (Corning) coated with 30 μl matrix gel (diluted 1:8) (Sigma-Aldrich, St. Louis, MO, USA), and the invasive analysis was performed. Medium containing 10% FBS was added to the lower compartment and cultured for 48 h at 37° C. Non-invasive cells were detached from the top chamber of the Transwell inserts using cotton swabs, and the invasive cells at the bottom were fixed in 100% methanol for 30 min. After air drying, cells were stained with 0.5% crystal violet (Sigma-Aldrich, St. Louis, MO, USA), imaged and counted under an optical microscope. Normalized invasion cell number = actual invasion cell number/cell growth rate.

### Colony formation assay

Trypsin-treated cells were placed on a 6-well plate with 300-500 cells per well and maintained in Polylex medium containing 10% FBS for 2 weeks. The colonies were placed in methanol for 30 min and 500 μl 0.5% crystal violet was added to each well for 30 min. Visual counting was performed.

### Wound-healing assay

For the wound healing experiment, a fused cell monolayer was grown in a 6-well plate to create a uniform acellular wound zone. A 10-μl pipette tip was used to scratch the monolayer slightly. After wound formation, a 0-h image was taken using an inverted apparent fluorescence microscope to better compare and determine the wound healing rate 24 h after scratching. The migration area between the dotted lines was measured and confirmed in ImageJ and normalized to the migration area of control cells.

### Immunohistochemistry (IHC)

We previously described the experimental immunohistochemical process in detail [39]. Briefly, the collected human breast cancer and xenograft tumor samples were embedded in paraffin, and 4-μm thick sections were dewaxed in xylene and rehydrated in a graded ethanol series. Sections were incubated with 0.3% H2O2, and the antigen was recovered in citrate buffer. The primary antibody was visualized using an HRP-labeled secondary antibody (Gene Tech, Shanghai) and diaminobenzidine (DAB). The sections were stained with hematoxylin, dehydrated in ethanol, cleared in xylene, and covered with resin. Antibodies against GABARAP (Proteintech, 11010-1-AP, dilution 1:100), E-cadherin (Proteintech, 20874-1-AP, dilution 1:4000), p-mTOR (Ser2448; Affinity, AF3308, dilution 1:50), matrix metalloproteinase (MMP) 2 (Proteintech, 10373-2-AP, dilution 1:200) and MMP14 (Proteintech, 14552-1-AP, dilution 1:200) were used for the IHC analyses.

H-score was calculated as 0 for no staining, 1 for weak positive (light yellow staining), 2 for medium positive, and 3 for strong positive (brown staining). The positive cells were divided into four groups with percentages of < 5%, 5% - 25%, 26-50%, 51% - 75% and > 75% representing tumor cell staining of grade 0, grade 1, grade 2 and 3, and grade 4. The positive grade was multiplied by the score as 0 is negative (-), 1-4 weak positive (+), 5-8 positive (+ +), and 9-12 strong positive (+ + +).

### Western blotting

Cells were lysed using RIPA lysis buffer (Solarbio) containing protease inhibitors (Beyotime) and a phosphorylase inhibitor cocktail (Roche) to obtain protein, and the contents were 990 μl RIPA lysis buffer + 10 μl PMSF + 100 μl phosphorylase inhibitor. A BCA Protein Assay Kit was used to confirm the concentrations of protein separated using 10% SDS-polyacrylamide gel electrophoresis and transferred to PVDF membranes. The membranes were blocked with 5% BSA blocking reagent for 1 h at room temperature (RT) and incubated with primary antibodies overnight at 4° C. The membranes were washed and incubated for 1 h at RT with secondary antibodies. The proteins were analyzed using the ECL Plus kit. The following antibodies used included at a dilution of 1:1000: GABARAP (Abcam, ab109364), E-cadherin (Abcam, ab40772), N-cadherin (Abcam, ab76011), vimentin (Proteintech, 10366-1-AP), MMP2 (Abcam, ab110186), MMP14 (Abcam, ab3644), AKT (Abcam, ab179463), p-AKT (Bioworld Technology, Ser473, BS4007), mTOR (Abcam, ab2732), p-mTOR (Ser2448; 5536), p70S6K (Proteintech, 14485-1-AP), and p-P70S6K (Thr389; 9234), MEK1/2 (8727), p-MEK1/2 (Ser217/221; 9154), ERK1/2 (4695), p-ERK1/2 (Thr202/Tyr204: 4370), IKK-β (8943), p-IKK-β (2078), IκBα (4812), and p-IκBα (2859) from Cell Signaling Technology. β-actin was used as an internal control (ZSGB-BIO, TA-09, dilution 1:1500).

### Nude mice tumor xenograft model

Female BALB/C nude mice, 4 to 5 weeks old, were obtained from Beijing Vital River Laboratory Animal Technology Co., Ltd. and fed in the Animal Center of the Affiliated Tumor Hospital of Harbin Medical University. UACC-812 / vector control or UACC-812 / GABARAP shRNA cells (5×10^6^ cells in 100 μl PBS) were injected subcutaneously into the left side in each group (n=10). After the formation of a palpable tumor, the mice carrying UACC -812 cells were randomly divided into two subgroups (n=5). LY294002 (75 mg/kg) or sterile water was injected intraperitoneally twice weekly for 3 weeks. The tumor volume was monitored using vernier calipers every 5 days for 3 weeks. On the last day of the experiment, the formula (L×W^2^) / 2 was used to calculate the tumor weight after resection, where L was the length and W was the width. All of the mice were euthanized, and the tumor tissue was partially anchored in formalin and immersed in paraffin for IHC analysis. All relevant experiments received approval from the animal protection and use Committee (IACUC) of the university mentioned above and were performed in accordance with NIH guidelines for the care and use of laboratory animals.

### Statistical analysis

Statistical analyses were performed using SPSS 22.0 and GraphPad Prism software. All experiments were performed at least three times. The data are expressed as means ± standard deviation (SDs), and disparities between two groups were analyzed using Student’s t tests and the χ2 test, and survival was analyzed using Kaplan Meier analysis and the log-rank test. The UALCAN database (http://ualcan.path.uab.edu/index.html) and Oncomine Database (https://www.oncomine.org/resource/login.html) were used to download the cancer genome map of invasive breast cancer (tcga-brca) cohort data. The relevant data sets were used to detect GABARAP mRNA expression and survival analysis of breast cancer. Double tailed P < 0.05 was considered significant.

## Supplementary Material

Supplementary Figure 1

Supplementary Tables
