# Supplementary material for: GABARAP suppresses EMT and breast cancer progression via the AKT/mTOR signaling pathway
Source: Aging (Albany NY). 2021 Feb 11;13(4):5858–74. doi: 10.18632/aging.202510 (PMC7950252; doi:10.18632/aging.202510)
Supplement: Supplementary Figure 1 [file aging-13-202510-s001.pdf]

SUPPLEMENTARY FIGURE

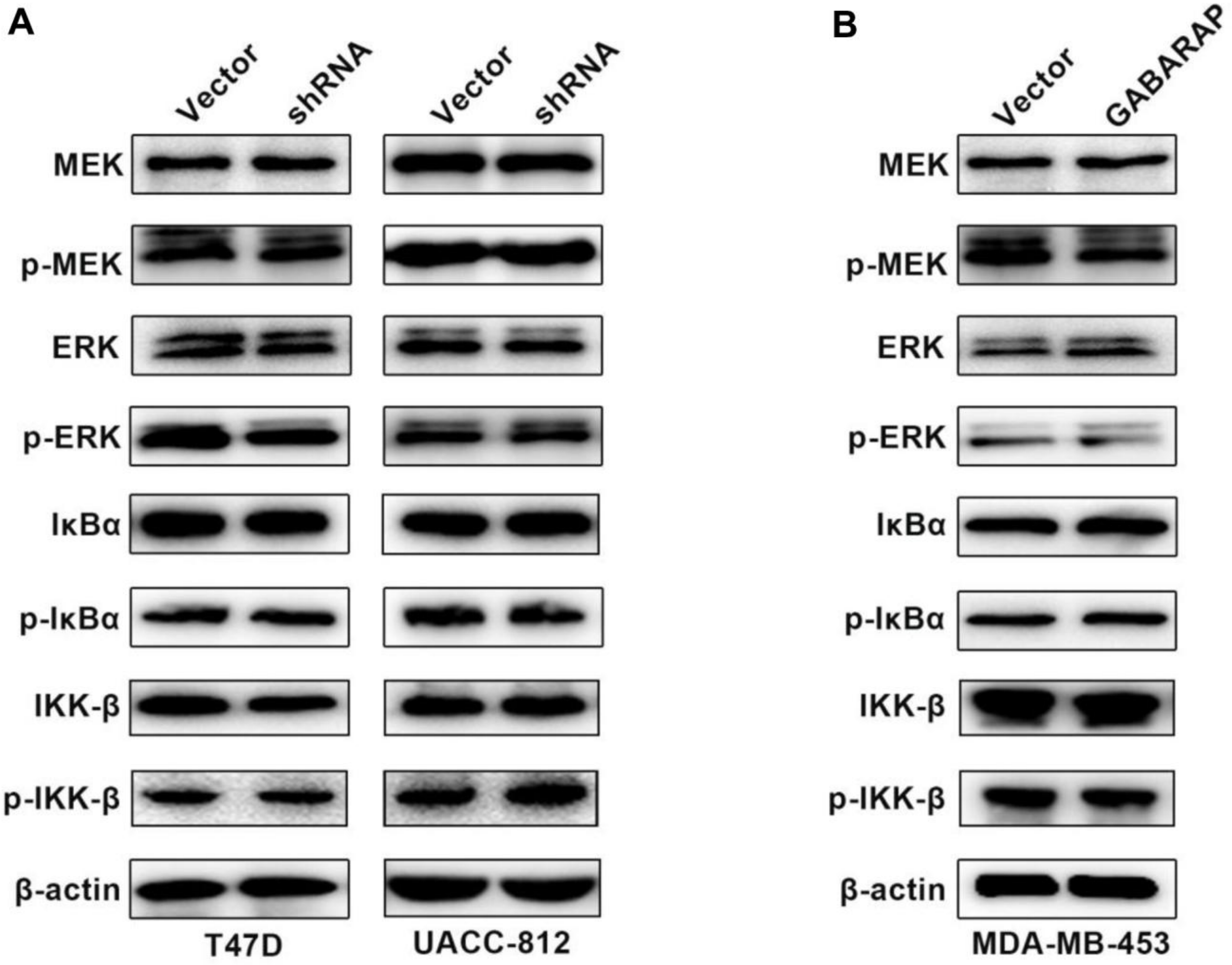

Supplementary Figure 1. Downregulation or upregulation of GABARAP did not activate the NF-κB or ERK/MAPK signaling pathways. (**A**) Western blot analyses were used to detect the expression levels of p-IKK-β, IKK- β,p-IκBα, IκBα, p-ERK, ERK, p-MEK, and MEK in T47D-vector, T47D-shRNA, UACC-812-vector, and UACC-812-shRNA cells. (**B**) Western blot analyses were used to detect the expression levels of p-IKK- β, IKK- β, p-IκBα, IκBα, p-ERK, ERK, p-MEK, and MEK in MDA-MB-453-vector and MDA-MB-453-GABARAP cells.
