# Supplementary material for: GABARAP suppresses EMT and breast cancer progression via the AKT/mTOR signaling pathway
Source: Aging (Albany NY). 2021 Feb 11;13(4):5858–74. doi: 10.18632/aging.202510 (PMC7950252; doi:10.18632/aging.202510)
Supplement: Supplementary Tables [file aging-13-202510-s002.pdf]

SUPPLEMENTARY TABLES

Supplementary Table 1. GABARAP protein expression levels in different pathological types.

	Staining intensity		Total	P value*
	Negative(%)	Positive(%)		
Non-tumor tissue	8 (33.33)	16(66.67)	24	0.006
DCIS	31(64.58)	17(35.42)	48	
IDC	60(68.970	27(31.03)	87	

Supplementary Table 2. Correlations between GABARAP with clinicopathological features in 87 breast cancer patients.

Variable	NO. (n=87)	GABARAP expression		P value
		Negative (%)	Positive (%)	
Age (year)				
≤35	6	2 (33.3)	4 (66.7)	>0.99
>35	81	23 (28.4)	58 (71.6)	
Tumor size (cm)				
<2	14	2 (14.3)	12 (85.7)	0.025
2-5	68	28 (41.2)	40 (58.8)	
>5	5	4 (80.0)	1 (20.0)	
Lymph nodes metastasis				
Negative	51	8 (15.7)	43 (84.3)	0.023
Positive	36	14 (38.9)	22 (61.1)	
TNM stage				
I	7	1 (14.3)	6 (85.7)	0.001
II	64	21 (32.8)	43 (67.2)	
III	16	13 (81.3)	3 (18.7)	
Histological grade				
I; II	42	15 (35.7)	27 (64.3)	0.019
III	45	28 (62.3)	17 (37.7)	
ER status				
Negative	28	11 (39.3)	17 (60.7)	0.039
Positive	59	11 (18.6)	48 (81.4)	
PR status				
Negative	38	13 (34.2)	25 (65.8)	0.092
Positive	49	9 (18.4)	40 (81.6)	
Her-2 status				
Negative	47	10 (21.3)	37 (78.7)	0.459
Positive	40	12 (30.0)	28 (70.0)	
Ki-67 status				
Negative	43	8 (18.6)	35 (81.4)	0.218
Positive	44	14 (31.8)	30 (68.2)	
P53 status				
Negative	60	17 (28.3)	43 (71.7)	0.330
Positive	27	5 (18.5)	22 (81.5)	

Note: A chi-square test was used for comparing groups between low and high GABARAP expression. TNM: Tumor lymph node metastasis. *$p<0.05$ was considered significant.
